# Editorial Notes

**Published:** 1935

**Authors:** 


### Editorial Notes

**Dr. Conrad Fitz-Gerald of Newfoundland.**

MESSRS. ARROWSMITH announce that a Biography of Dr. Conrad Fitz-Gerald of Fortune Bay, Newfoundland, is now being printed. His name is known to some of our readers as being the senior student on the roll of the Bristol Medical School, which he entered as long ago as 1866. A Foreword by one of his old friends speaks of the "brilliant exploits of this grand old gentleman, Dr. Fitz-Gerald, as a surgeon and navigator on the south coast of Newfoundland. He is beloved by the vast host of his patients, many of whom he attended at the risk of his life, beating up and down that treacherous, cruel and rocky coast in his good ship *Albatross*, manned entirely by himself as crew. Often he experienced raging gales and mountainous seas on a lee coast, and yet safely brought his good ship through these hard and desperate storms to safe anchorage in some friendly harbour, of which he seemed to have an uncanny knowledge concerning their tides and soundings. Often have I known him during the winter months battle his way on snow-shoes across this land of desolation against blizzards and heavy snow-storms to reach some isolated spot that the patient might have the advantage of his skill in medicine or surgery."

His grandson has gleaned from him the history of his life in England, and his travels round the world, which he is now publishing for private circulation. If any of our readers wish to obtain a copy of this thrilling account of seamanship and medical practice, orders may be sent to Messrs. J. W. Arrowsmith Ltd., 11 Quay Street, Bristol. In these days, when doctors are accustomed to driving to their patients in fool-proof cars on modern highways, the account of Dr. Fitz-Gerald's professional rounds, sailing a seven-ton yacht round the tempestuous coast of Newfoundland, will arouse more feelings of contentment than envy. Fitz-Gerald's life is an outstanding example of how a man may achieve complete mastery in two very different callings. We are glad to know that as the sheets are in the press he enjoys such health that he could say of himself, "One of my legs is eighty-six, but the rest of me is fifty."

**Bristol Royal Infirmary Nurses' League News Letter.**

WE are interested to hear that a Bristol Royal Infirmary Nurses' League has been formed to keep in touch with former Bristol Royal Infirmary nurses. The first meeting was held in London in 1933. A news letter has just been issued which is very capably edited, and is really a most interesting document. It describes recent changes at the Infirmary, gives personal news, and in particular includes a most remarkable article by Miss C. F. Hill, in which she relates her experiences. The Infirmary in those days was evidently a terrible place. The nurses had sometimes to sleep on the floor, or in dark, unventilated cubicles infested with bugs. They were half-starved, had nowhere to spend their off-duty time, and had to learn the theory of their profession by what they could pick up from friendly medical students. The article is so interesting and informing that it deserves a very wide publicity.

**Cricket Challenge Cup.**

WE learn that the Staff of the Bristol General Hospital has presented a Challenge Cup to be held by the winners of the annual cricket match between the Royal Infirmary and the General Hospital "to foster the excellent relations that exist between the two Institutions." This generous gift should serve to increase the keen enthusiasm which the inter-hospital cricket match always arouses.

